# Health literacy in patients with chronic hepatitis B attending a tertiary hospital in Melbourne: a questionnaire based survey

**DOI:** 10.1186/1471-2334-14-537

**Published:** 2014-10-23

**Authors:** Tanya FM Dahl, Benjamin C Cowie, Beverley-Ann Biggs, Karin Leder, Jennifer H MacLachlan, Caroline Marshall

**Affiliations:** Victorian Infectious Disease Service (VIDS), Royal Melbourne Hospital, Grattan St, Parkville, 3050 Victoria Australia; Department of Medicine, University of Melbourne, Parkville, 3050 Victoria Australia; Epidemiology Unit, Victorian Infectious Diseases Reference Laboratory, Grattan St, Parkville, 3050 Victoria Australia; Department of Epidemiology and Preventive Medicine, Monash University, Melbourne, 3004 Victoria Australia

**Keywords:** Chronic hepatitis B, Immigrant health, Health literacy, Transmission, Management

## Abstract

**Background:**

Current estimates suggest over 218,000 individuals in Australia are chronically infected with hepatitis B virus. The majority of these people are migrants and refugees born in hepatitis B endemic countries, where attitudes towards health, levels of education, and English proficiency can be a barrier to accessing the Australian health care system, and best managing chronic hepatitis B. This study aimed to assess the knowledge of transmission and consequences of chronic hepatitis B among these patients.

**Method:**

A prospective study was conducted between May and August 2012. Patients with chronic hepatitis B were recruited from three Royal Melbourne Hospital outpatient clinics. Two questionnaires were administered. *Questionnaire 1*, completed during observation of a prospective participants’ consultation, documented information given to the patient by their clinician. After the consultation, *Questionnaire 2* was administered to assess patient demographics, and overall knowledge of the effect, transmission and treatment of hepatitis B.

**Results:**

55 participants were recruited. 93% of them were born overseas, 17% used an interpreter, and the average time since diagnosis was 9.7 years.

Results from *Questionnaire 1* showed that the clinician rarely discussed many concepts. *Questionnaire 2* exposed considerable gaps in hepatitis B knowledge. Few participants reported a risk of cirrhosis (11%) or liver cancer (18%). There was a high awareness of transmission routes, with 89% correctly identifying sexual transmission, 93% infected blood, and 85% perinatal transmission. However, 25% of participants believed hepatitis B could be spread by sharing food, and over 50% by kissing and via mosquitoes. A knowledge score out of 12 was assessed for each participant. The average score was 7.5. Multivariate analysis found higher knowledge scores among those with a family member also diagnosed with chronic hepatitis B and those routinely seeing the same clinician (p = 0.009 and p = 0.002, respectively).

**Conclusion:**

This is the largest Australian study assessing knowledge and understanding of the effect, transmission, and treatment of hepatitis B among chronically infected individuals. The findings highlight the knowledge gaps and misconceptions held by these patients, and the need to expand education and support initiatives.

**Electronic supplementary material:**

The online version of this article (doi:10.1186/1471-2334-14-537) contains supplementary material, which is available to authorized users.

## Background

Hepatitis B virus (HBV) is one of the most common chronic infections affecting up to 350 million people worldwide [1]. It is the second most important known human carcinogen after tobacco [1, 2] and was estimated to have resulted in the deaths of 786,000 people in 2010 [3].

The prevalence of chronic hepatitis B (CHB) in Australia has been estimated at approximately 1% of the population, affecting over 218,000 individuals in 2011 [4]. The majority of these are migrants from endemic areas, or Aboriginal and Torres Strait Islander people.

It is estimated that the increasing CHB prevalence in Australia, predominantly attributable to migration, will result in a two to three fold increase in premature mortality from CHB-related liver cancer by 2017 [5, 6], with liver cancer now the fastest increasing cause of cancer deaths in Australians [7]. Early diagnosis and appropriate management mitigates negative outcomes, including reducing the risk of liver cancer [8, 9] and reversing cirrhosis [10].

Recent migrants from non- English speaking hepatitis B endemic countries may have low health literacy [11], which reduces their capacity to navigate the Australian health care system and is a barrier to comprehending concepts such as liver disease, viral transmission, and antiviral treatment. Combined with fear of stigma, complications and misconceptions, this can be stressful and debilitating [12] and results in suboptimal management for the individual and ongoing transmission to susceptible contacts [13].

Improving understanding of hepatitis B in priority populations and enhancing access to diagnosis and care are key recommendations of Australia’s Second National Hepatitis B Strategy 2014-2017 [14]. Regular monitoring including clinical reviews and appropriate investigations are essential for all people with CHB [15]. Reducing alcohol consumption [16] and smoking cessation also reduce progression to advanced liver disease [17], as does recognition of symptoms associated with disease progression. Those receiving antiviral treatment need to be aware of the potentially indefinite duration of treatment, and that non-adherence allows viral rebound and/or resistance leading to adverse health outcomes [18]. Initiatives to improve testing and vaccination for contacts, and increased monitoring, treatment and educational options for those infected, are essential for reducing the burden of CHB [15].

In light of the increasing burden of CHB in Australia, and the identified need to improve both engagement with and care delivery for people living with CHB, this study aimed to understand current patients’ knowledge and understanding of the transmission, complications and treatment of CHB, and to investigate factors associated with the degree of knowledge in these individuals, in order to guide improvements in clinical and educational initiatives for the growing number of Australians living with CHB.

## Methods

### Patient cohort

Adult patients with CHB were recruited from three clinics at the Royal Melbourne Hospital from May to August 2012. Two of these clinics saw only patients with a diagnosis of chronic viral hepatitis, and the third clinic was an immigrant health clinic, seeing a range of infections. Some patients routinely saw the same physician, while others were seen by physicians based upon order of arrival at the clinic. All patients diagnosed with CHB aged over 18 years were eligible for participation. A sample size of 50 was determined based on the time available for the study, the likely number of patients able to be recruited in this time period, and the number of participants required to obtain significant results in a similar study of patients with latent tuberculosis conducted at the Royal Melbourne Hospital [19].

Two survey forms were developed and administered by the one independent researcher (T.D). A piloting period was conducted and some questions altered prior to study commencement. Verbal consent was obtained following an explanation of the study and the entirely voluntary nature of participation.

*Questionnaire 1* recorded information given to participants during their follow-up consultation, as observed by the researcher. *Questionnaire 2* was administered immediately following the consultation, documenting factors including age, education, country of birth, and if an interpreter was used. It also examined patient knowledge with specific open and closed questions covering transmission and consequences of CHB, and possible treatment. The same interpreter (if required) was used during the consultation and administration of the questionnaire. Interviews were conducted by the one researcher to ensure consistency and accuracy of administration. The questionnaire was read out loud to participants to avoid literacy bias.

### Data analysis

A knowledge score was derived based on the answers to five questions in *Questionnaire 2* reflecting core knowledge of CHB to provide an overall score out of 12. Points were allocated as follows: one point for identifying hepatitis B as their reason for coming to the clinic, one point for identifying their liver as the affected organ, and another for an outcome such as cirrhosis, liver damage or HCC in their answer of how HBV can affect the body. Half a point was awarded for each correct response to the ten yes or no questions on how HBV can be transmitted. When asked the reason for being treated or not being treated, patients were given one point for identifying the lack or presence of liver damage, and one for mentioning a high or low viral load. Finally, two points were awarded for identifying either reducing alcohol consumption or eating well as factors their doctor mentioned to improve liver health. Data were analysed using Stata v10 (College Station, Texas). Univariate analysis (Wilcoxon rank-sum test and linear regression) was conducted to detect associations between knowledge scores and demographic characteristics. Given the large number of variables assessed relative to sample population size, a parsimonious multivariate model was constructed, including only those variables that showed relatively high degrees of association with knowledge score (in this case those with a p value of less than 0.05 on univariate analysis).

To eliminate any ordinal variation in the model, a forwards and backwards stepwise multivariate regression model was constructed to estimate variables associated with knowledge score (analysed as a continuous variable). The resulting significance of output was determined at p ≤ 0.05 with a coefficient produced for each variable. The coefficient describes a multiplicative factor relating the knowledge score and socio-demographic variable; for example, in the case of gender the coefficient would denote the difference in knowledge score between females and males. In terms of age, it would denote the change in knowledge score per year. All other data were analysed descriptively.

### Ethics

This project was approved by the Melbourne Health Human Research Ethics Committee as a quality assurance proposal (approval number QA2012029) and is in compliance with the Helsinki Declaration. All participants gave informed consent.

## Results

### Concepts discussed during consultation with clinician

Questionnaire 1 was completed during standard patient follow-up consultations. No participants were attending a first appointment, and doctors were not given a standard script but were observed in their routine practice.

The risks of sexual transmission, blood transmission and perinatal transmission were discussed in 14.2%, 8% and 8% of consultations respectively. Vaccination of family and sexual contacts was also discussed in 17% and 8% of consultations respectively. Possible life-style changes were raised including reducing alcohol (31%), smoking cessation (19%), and weight loss (7%).

### Participants (as assessed via Questionnaire 2)

Of the 58 patients invited to participate, 55 were recruited for the study. Incomplete knowledge data were collected from four during the piloting period, and these results were consequently only included in relevant descriptive analyses.

**Figure 1:**
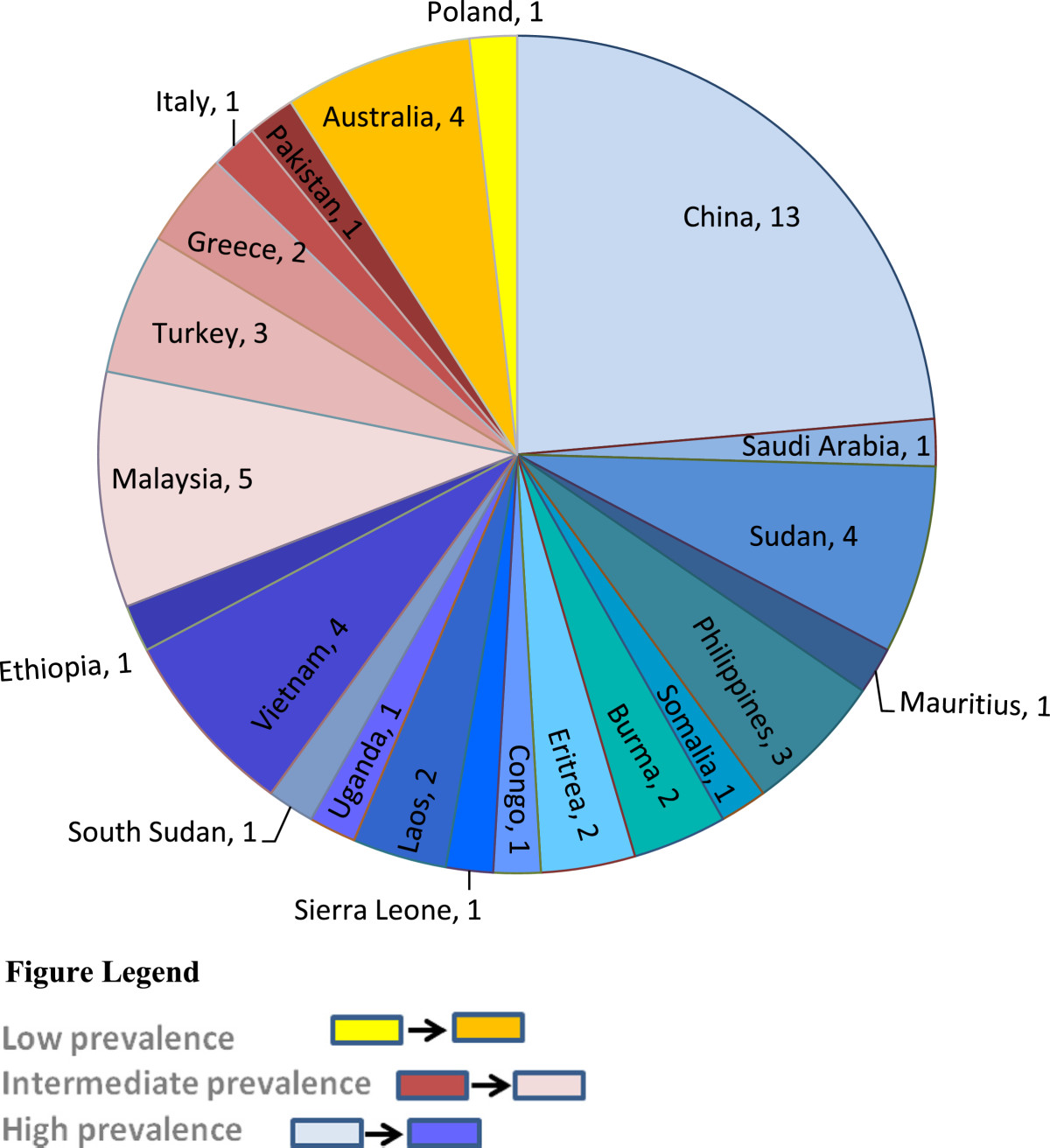
**Country of birth and HBV endemicity of the study group (n = 55).**

The socio-demographic characteristics of the study group are shown in Figure 1 and Table 1. The distribution of countries of birth of the patients is shown in Figure 1.

**Table 1:** **Profile of the study group (N = 55)**

Study group characteristics		No.	% of cohort
Gender	Male	37	67
	Female	18	33
Born in Australia	Yes	4	7
	No	51	93
Time in Australia (Excluding those born in Australia n = 51)	< 2 years	0	0
	2-5 years	9	18
	5-10 Years	19	37
	>10 Years	23	45
Lived in a refugee camp	Yes	14	25
	No	41	75
Educational level	Primary	4	7
	Secondary	20	37
	Tertiary	31	56
Use of an interpreter during consultation	Trained	6	11
	Family/Friend	4	7
	None	45	82
English Literacy	Yes	42	76
	No	13	24
Preferred language literacy	Yes	54	98
	No	1	2

Over half (56%) had completed tertiary education with all but one participant literate in their preferred language. The participant unable to read in either English or their preferred language was also the only participant not to have a family member or friend literate in either language. Of those not able to read English, all others had an English-literate family member or friend. The average time since diagnosis was 9.7 years, with over half having a family member or friend also diagnosed with CHB.

### Participant knowledge

Questionnaire 2 responses are shown in Table 2.

**Table 2:** **Participant responses to Questionnaire 2 assessing HBV knowledge (N = 48)**

Knowledge questions	Answer	No.	%
**Why are you coming to this clinic?**	Hepatitis B	43	78
	Hepatitis	3	5
	Other (incorrect/unsure)	9	16
**What causes hepatitis B?**	Concept of Virus	19	44
	Concept of something attacking liver/infection	3	7
N = 43- Significant prompting^†^			
	Incorrect	4	9
	Unsure	17	40
**How can hepatitis B affect your body?**	Affects the liver	36	65
	Jaundice	2	4
	Fatigue	5	9
Multiple answers allowed	Asymptomatic	7	13
	Liver damage, cirrhosis	6	11
	Liver cancer	10	18
	Incorrect	3	5
	Unsure	14	24
	Emotional/stigma	5	9
**Can Hepatitis B be spread to someone else?**	Yes	50	91
	No	2	4
	Unsure	3	5
**How can hepatitis Be spread to someone else?**	Sexual transmission	27	49
	Blood contact	35	64
Unprompted^‡^			
Multiple answers possible			
	Needle contamination	7	13
	Perinatal	9	16
	Medical contamination	5	9
	Other- Bodily fluids, Nail clipping, Saliva	13	24
	Incorrect	4	7
**Prompted Transmission: No. % correct**	Sharing food	41	75
	Sexual Contact	49	89
	Contact with infected blood	51	93
	Kissing	26	47
	From mother to child during birth	45	82
	Unclean needles	48	87
	Breathing	44	80
	Mosquitoes	23	42
	Blood transfusion	50	91
	Unclean medical equipment	47	85
**Why are you being treated/starting treatment for Hepatitis B?** N = 31	Mentioned virus only	10	32
	Mentioned liver damage only	11	35
	Mentioned both	2	7
	Unsure/Incorrect	8	26
**Those on treatment** N = 29 (53% of cohort)	Correctly named medication	16	55
	Indefinite antiviral course	22	76
	Never skip dose	21	72
**Do you know why you are not being treated for Hepatitis B?** N = 24 (44% of cohort)	Mentioned virus	8	33
	Mentioned liver damage	2	8
	Mentioned both	2	8
	Unsure/Incorrect	10	42
	Other	2	8
**Is there anything you can do to improve your Hepatitis B?** Multiple answers allowed N = 51	Yes	43	84
	Reduce alcohol	24	47
	Stop smoking	7	14
	Lose weight/exercise	10	20
	Treatment	5	10
	Other- Iron and Vit D supplement, being healthy	2	4
	Incorrect	7	13
**Do you feel comfortable telling family and household members about this condition?**	Yes	51	93
	No	4	7
**Do you feel comfortable telling friends and people you work with?**	Yes	19	35
	No	36	65
**Do you usually understand what the doctor tells you about Hepatitis B?**	Yes	52	95
	No	3	5
**Do you think the explanation of Hepatitis B by your doctor was adequate?**	Yes	50	91
	No	5	9
**Other medical conditions**	Yes	36	65
	No	19	35

^†^This question was not answered correctly during the piloting period, therefore these participants were not included in the results for this question. All the other participants received significant prompting such as: “What is hepatitis B in your body? Is it a germ, or microbe, or something else?” The results of this question were not included in the knowledge assessment, as the question was often misunderstood and therefore appears not to be a good assessment of knowledge.

^‡^For transmission questions, patients were first asked if HBV could be spread to someone else, and if yes, how it could be spread. They were then prompted as to whether HBV can be spread by sharing food, sexual intercourse, and so on.

To assess transmission knowledge, participants were first asked how HBV could be spread, and then were asked to answer “yes” or “no” to prompted transmission options. All but five participants were aware that HBV could be spread to someone else, with those five being confused about transmission, some answering “yes” or “unsure” to all prompted transmission routes offered.

Over half of the participants were receiving treatment for their CHB, and of these, 55% could name the medication. 76% were aware that treatment duration would likely be life-long.

56% of participants indicated they would like to receive more information. Of those declining more information, some stated they already had enough (most often online), and some just said they did not did want additional information. Participants not using an interpreter were less likely to want more information (53%) compared to those who used one (70%). Of those wanting more information the main preferences were for written or online information both in English and a range of primary languages.

Anecdotally, many patients were keen to share the negative impact that hepatitis B had upon their lives, with one participant saying that it “had destroyed (his) life”. This is further reflected by the fact that 65% of participants were not comfortable discussing their CHB diagnosis with friends and work colleagues, as well as the 7% not comfortable telling their family.

### Overall knowledge score

The knowledge score represents responses to five questions that assessed overall knowledge of HBV. The average knowledge score was 7.5 out of a possible 12 points. Only one participant answered all questions correctly with 52% of the study group scoring below 8 and the lowest score being 2.5.

Univariate analysis correlated 16 participant demographic variables with participant knowledge scores (Table 3). All demographic variables that were significantly associated with CHB knowledge are marked with an asterisk. Use of an interpreter and having completed tertiary education approached significance (p = 0.053 and p = 0.059 respectively) and were therefore also included in the multivariate model, as was participant age.

**Table 3:** **Univariate analysis of associations between knowledge scores and demographic characteristics of the study cohort**

Sociodemographic characteristics		No. (%)	Median knowledge score	Interquartile range	p-value
**Gender**	Male	35 (67)	7.5	5-8.8	0.0268**
	Female	16 (33)	8	7.75-9.5	
**Interpreter used**	None	42 (82)	8	6-9	
	Trained	5 (9)	7	3-8	0.059
	Family/Friend	4 (8)	7.75	5.25-9.25	0.69
**English Literacy**	Yes	39 (76)	6.8	4.25-8	0.045**
	No	12 (24)	8	6-9.5	
**Lived in a refugee camp**	Yes	13(25)	8	7-8.5	0.63
	No	38 (75)	7.5	5.5-9	
**Time in Australia**	Continuous	-			0.65
	<2 years	0			
	2-5 years	8 (16)	7.25	5.25-9.5	
	5-10 Years	19 (35)	7.5	6-9.5	0.97
	>10 Years	24 (49)	8	7.25-8.75	0.79
**Educational level**	Primary	4 (8)	5.75	3.75-8.25	
	Secondary	19 (37)	7.5	5-8.5	0.48
	Tertiary	13 (25)	8	7.5-9.5	0.05**
	Tertiary +	15 (29)	8	6-10	0.14
**Clinic**	Public hepatitis	22	8	5.5-9	
	Immigrant and refugee	7	7	4.5-8	0.34
	Privatised hepatitis	22	7.5	6-9.5	0.24
**Time since diagnosis**	< 1 year	3 (6)	5.5	5-7.5	
	2-5	18 (35)	7.5	6-8	0.45
	6-15	17 (33)	8	6-10	
	>16	13 (25)	8	7-9	0.22
**Country diagnosed**	Australia	41 (80)	7.5	5.5-8.5	0.19
	Other	10 (20)	8.5	6.5-10.5	
**Seen clinician previously n = 44**	Yes	27 (61)	8	7-9.5	0.010**
	No	17 (39)	6	4.5-8	
	No. of years seeing clinician				0.003**
**Know anyone else with HBV**	No-one	24 (47)	7.3	5.25-8	
	Family	20 (39)	8.3	7.75-9.5	0.007**
	Friends	3 (6)	8	4.5-10	
	Both	2 (4)	6.3	4.5-8	0.73
	Not specified	2 (4)	5.8	4-7.5	
**Family comfortable**	Yes	47(92)	7.8	6-8.75	0.94
	No	4(8)	8	6-9	
**Friend comfortable**	Yes	32(63)	8	7.25-9.25	0.049**
	No	19(37)		4.5-8	
**Age**	Continuous				0.21
	Under 30	11	8	6-10	
	30-45	21	8	7-9	0.75
	46-60	11	7.5	6-9.5	0.96
	61 and over	8	6.8	4.75-8.25	0.19

**Significant at ≤ 0.05.

### Multivariate analysis

Results of the multivariate analysis are shown in Table 4. Backwards and forwards step-wise regression suggested that having a family member with CHB and having seen the same clinician more than once were associated with a better knowledge score.

**Table 4:** **Multivariate regression model of variables associated with knowledge score**

Socio-demographic characteristics	Coefficient	95% confidence interval	P -value
Know both family and friends diagnosed with HBV	-3.26	-6.96-0.42	0.081
English literate	0.96	-0.32-2.24	0.14
Know family member diagnosed with HBV	1.54	0.40-23.68	0.009**
Having seen the doctor before	1.91	0.78-3.047	0.002**

R^2^ = 0.42 (i.e. 42% of the variability seen within the knowledge score is explained by this model).

**Significant at ≤ 0.05.

## Discussion

A limited number of studies have been conducted into the knowledge of people living with CHB regarding their condition [20–22]. This is the largest Australian study to investigate knowledge of CHB among patients attending specialist outpatient clinics. The majority of study participants (93%) were born overseas (62% from Asia, 22% from Africa, 7% from Europe and 9% from Oceania) and had a family member also diagnosed with CHB. This validates observations that the predominant burden of CHB is experienced by migrants from endemic countries where perinatal and early childhood acquisition is common, resulting in multiple affected family members. It also emphasises the cultural diversity seen among individuals with CHB, and the consequential accommodation required by the health system [23]. Knowledge of the effects and risks associated with CHB was low in over half the participants, and there were major misconceptions regarding HBV transmission. There was significantly higher knowledge among individuals with a family member also diagnosed with CHB, and among patients routinely seeing the same doctor.

Important knowledge gaps were identified, for example, the majority of participants lacked understanding about the sequelae of CHB beyond its effect on the liver, with one answering that it affected the lungs. Although CHB is often asymptomatic, complications can occur, and patients should be aware of symptoms that could indicate progressive disease. Our findings contrast with a Malaysian study [24] where participants had a greater knowledge of symptoms such as jaundice and fatigue (55% and 54% respectively). However, unlike the present study, which used both open and closed questioning, the Malaysian study used only prompted yes/no questions, potentially limiting the comparability of these results.

The majority (89%) of participants were aware that HBV can be sexually transmitted, which is higher than the 79-80% reported in previous studies [24, 25]. Moreover, whereas only 6.8% of participants in the Malaysian study [24] knew that HBV was not spread by sharing food, 75% of patients questioned in the present study were aware of this. That HBV can be spread by sharing food and eating utensils is a common misconception, especially among Asian communities, where hepatitis A prevention strategies were promoted following outbreaks in China in 1988 [26]. As hepatitis A is spread via the faecal-oral route, prevention strategies included not sharing food or eating utensils, and this has since been confused with HBV transmission routes.

Over half of participants in our study believed HBV could be spread via kissing or mosquito bites, and 20% by breathing. Such misconceptions have been found by similar studies in other countries [27–31]. These misconceptions can have very deep and negative impacts on those affected, constituting an unnecessary and preventable burden. Dispelling these misconceptions is important for improving the quality of life of those infected [32, 33], and increasing awareness in the general community to reduce the stigma that arises from lack of knowledge.

Only four participants showed an understanding of the virus’s effect on the liver and how this related to their treatment status. During consultations terms such as viral load, and viral activity were commonly used, but we do not know if they were always understood by participants.

Some participants showed a passive approach towards their health, deferring to their doctor’s advice as their reason for treatment and appointment attendance. Passivity towards health has been correlated with low health literacy [34] and highlights the need for patient engagement as well as education, and this is particularly pertinent among recently arrived migrants who may have many competing priorities. It is important to note that the group interviewed represents those engaged with the healthcare system and attending specialist appointments. There are many individuals with CHB not engaged with the healthcare system, and more than 100,000 Australians estimated to be living with undiagnosed CHB [4], whose health literacy may be even lower.

The results of Questionnaire 1 suggest that patients are generally not provided with detailed information during ongoing monitoring of CHB. However, it is clear that some participants would benefit from repetition of important transmission and management information. While it could be assumed all participants were provided with extensive information upon diagnosis, it is possible that long-term retention of this information is not universal. Therefore, assumptions of knowledge may not be valid, irrespective of whether the cause is never having been provided with the information, or not recalling it.

Better knowledge was seen among those with a family member also diagnosed with CHB, possibly by providing a direct source of information following diagnosis. Having a relative with the same condition, especially if they have suffered the sequelae of liver dysfunction, may also promote communication, engagement, support and encouragement for regular monitoring [33]. Discussing the diagnosis with people sharing cultural and linguistic ties may also be conducive to better knowledge, although this could also be a source of the many myths regarding CHB infection [12]. Community-outreach programs based on providing culturally and linguistically salient information have been internationally recognised for effectively disseminating information among Hispanic/Latino communities in the United States [35].

Seeing the same the doctor has been shown to improve patient knowledge and management of chronic conditions such as asthma [35]. Our results validate this as positive associations with knowledge score were observed among patients having seen the clinician before (p = 0.02) and with increasing years seeing the same clinician (p = 0.003). A possible explanation is that with return visits, the treating clinician can incrementally educate the patient and reinforce previous discussions although more research in this area is needed. It also provides consistency and familiarity for the patient. This should be incorporated into clinical practice aimed at improving patient engagement with the healthcare system and reducing the number of patients lost to follow up.

During the study period, between 9%-31% of patients failed to attend their appointments (all clinic patients rather than only those with CHB). This provides an indication of the difficulty of monitoring and maintaining contact with patients who experience language barriers and may change residential address frequently. Wu et al [25] cited inconvenience as a significant barrier to healthcare access among 40% of participants with CHB, with after-hour clinics having higher attendance rates. The clinics in our study operated from 9 am-12.30 pm sometimes with considerable delays in seeing patients. The finding that the majority of patients stated that they did not feel comfortable disclosing the infection to their friends and colleagues highlights the potential difficulty of attending appointments during business hours.

This study had several limitations. As recruitment was from clinics based in a major Victorian hospital, it does not provide information about patients from other settings such as general practice or rural clinics. Our study group may also have had a slight bias towards those not using an interpreter, as on two occasions the interpreter was not able to complete the interview, preventing those patients from participating. Furthermore, patients attending these clinics are already engaged with the healthcare system, and may not be representative of the general population living with hepatitis B.

Patient acceptance of the research was high, with 55 of 58 invited agreeing to participate. However, with a number of variables assessed for impact on knowledge score, and considered for inclusion in multivariate analysis, this analysis may have lacked power to detect some factors truly associated with knowledge score (type II error).

## Conclusion

As HBV prevalence and attributable liver cancer incidence increases in Australia and other developed countries, health service provisions for people with CHB will need to improve and expand, and should involve education and support for those chronically infected and their families. There are many HBV patient resources, both online and in print, available in a number of priority languages (for example http://www.hepbhelp.org.au). Liaison nurses and community self-management programs also exist to support and educate this group. These resources are currently underused and it was one intention of this research to provide results that challenge clinicians to aim for improved engagement and continued education of this patient group, especially for long-term patients. These results also highlight the need to educate patients regarding common misconceptions in addition to actual transmission routes. A better understanding of CHB will improve compliance and monitoring and may reduce the significant morbidity, anxiety and stress surrounding CHB infection.

## Authors’ original submitted files for images

Below are the links to the authors’ original submitted files for images. Authors’ original file for figure 1

## Abbreviations

CHB: Chronic hepatitis B
HBV: Hepatitis B Virus.
