# Supplementary figures and images for: Health literacy in patients with chronic hepatitis B attending a tertiary hospital in Melbourne: a questionnaire based survey
Source: BMC Infect Dis. 2014 Oct 23;14:537. doi: 10.1186/1471-2334-14-537 (PMC4287473; doi:10.1186/1471-2334-14-537)

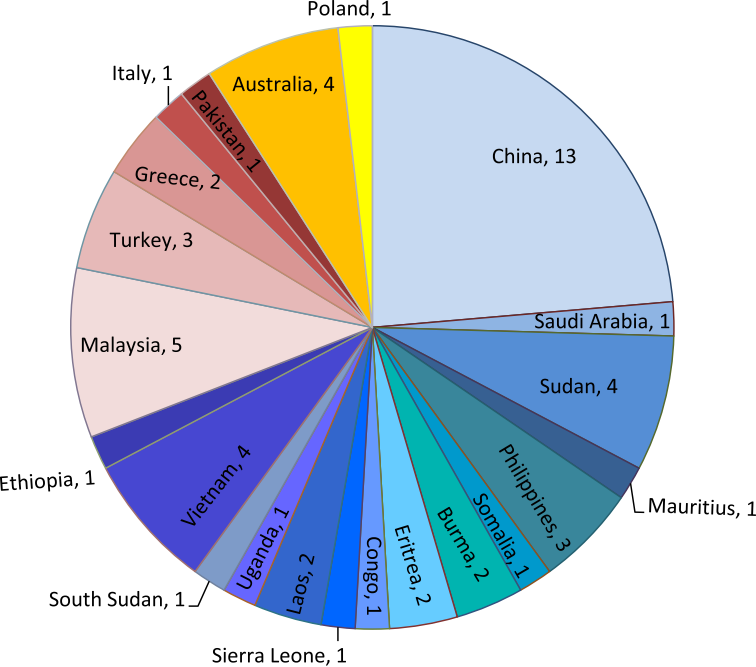

### Figure Legend

Low prevalence

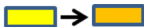

Intermediate prevalence

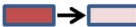

High prevalence

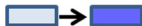

Supplement: Supplementary file 1 — Authors’ original file for figure 1 [file 12879_2014_4003_MOESM1_ESM.pdf]
